# Supplementary material for: Single-cell transcriptome dynamics of the autotaxin-lysophosphatidic acid axis during muscle regeneration reveal proliferative effects in mesenchymal fibro-adipogenic progenitors
Source: Front Cell Dev Biol. 2023 Feb 23;11:1017660. doi: 10.3389/fcell.2023.1017660 (PMC9996314; doi:10.3389/fcell.2023.1017660)
Supplement: Supplementary file 2 [file DataSheet2.PDF]

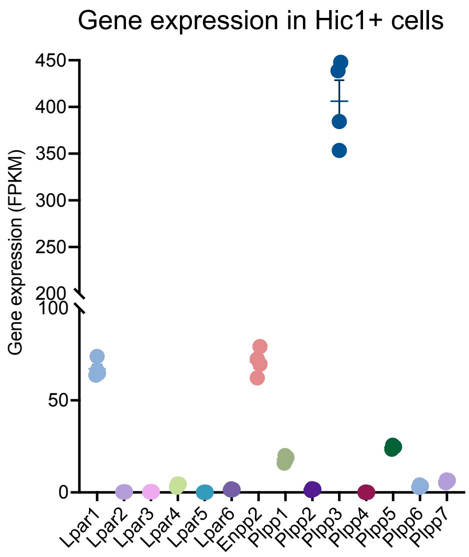

Supplementary figure 2. Differential gene expression of the LPAR-Autotaxin-Plpp network in mesenchymal stromal cells. (A) Quantification of Lpar, Enpp2, and Plpp transcript abundance (FPKM) in Hic1+ tdTomato expressing cells [Scott et al., 2019].
