# Supplementary material for: Single-cell transcriptome dynamics of the autotaxin-lysophosphatidic acid axis during muscle regeneration reveal proliferative effects in mesenchymal fibro-adipogenic progenitors
Source: Front Cell Dev Biol. 2023 Feb 23;11:1017660. doi: 10.3389/fcell.2023.1017660 (PMC9996314; doi:10.3389/fcell.2023.1017660)
Supplement: Supplementary file 5 [file Image9.pdf]

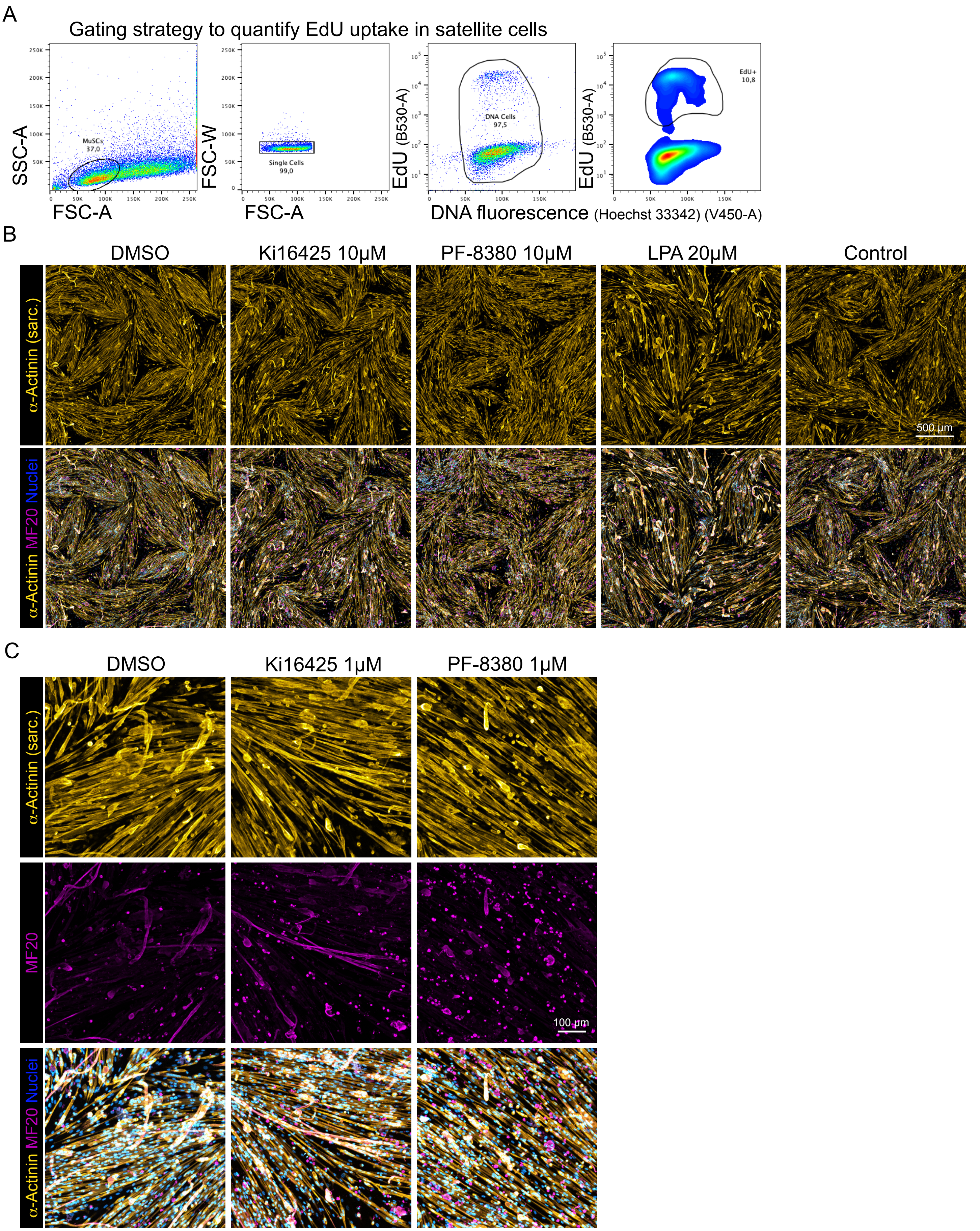

Supplementary figure 9. PF-8380 pharmacological inhibiton of ENPP2 impairs myotube differentiation of satellite cells. (A) Flow cytometry gating strategy to evaluate the % of EdU labelled satellite cells. (B) Representative laser confocal images of day 3 satellite cell-derived myotubes after the different treatments. α-Actinin staining in shown in *hot yellow*, nuclear staining in *hot blue*, and MF20 in *magenta*,. Scale bar: 500 μm. (C) Representative laser confocal images of day 3 satellite cell-derived myotubes after the different treatments at 1 μM. α-Actinin staining in shown in *hot yellow*, nuclear staining in *hot blue*, and MF20 in *magenta*,. Scale bar: 100 μm.
