# Supplementary material for: Single-cell transcriptome dynamics of the autotaxin-lysophosphatidic acid axis during muscle regeneration reveal proliferative effects in mesenchymal fibro-adipogenic progenitors
Source: Front Cell Dev Biol. 2023 Feb 23;11:1017660. doi: 10.3389/fcell.2023.1017660 (PMC9996314; doi:10.3389/fcell.2023.1017660)
Supplement: Supplementary file 6 [file DataSheet3.PDF]

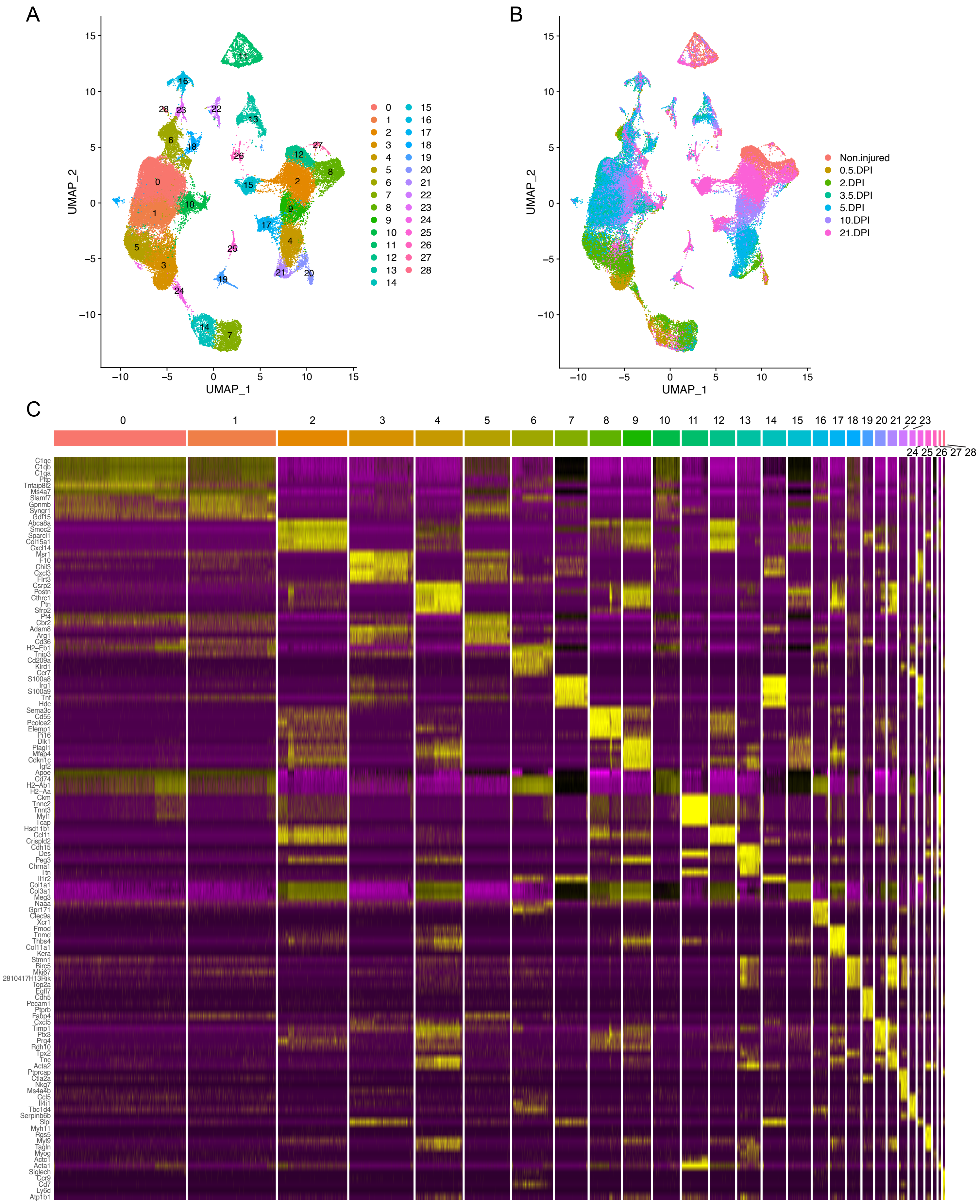

Supplementary figure 3. Initial scRNAseq seurat-based clustering analysis and conditions. (A) UMAP plot showing 29 distinct clusters across skeletal muscle homeostasis and regeneration. (B) UMAP plot showing distinct clusters based in non-injured or injured conditions. (C) Heat map plot of the 29 clusters shown in (A) and ordered based on the number of cells and the *top 5* expressed genes in each cluster subset.
