# Supplementary material for: Single-cell transcriptome dynamics of the autotaxin-lysophosphatidic acid axis during muscle regeneration reveal proliferative effects in mesenchymal fibro-adipogenic progenitors
Source: Front Cell Dev Biol. 2023 Feb 23;11:1017660. doi: 10.3389/fcell.2023.1017660 (PMC9996314; doi:10.3389/fcell.2023.1017660)
Supplement: Supplementary file 9 [file DataSheet8.PDF]

A

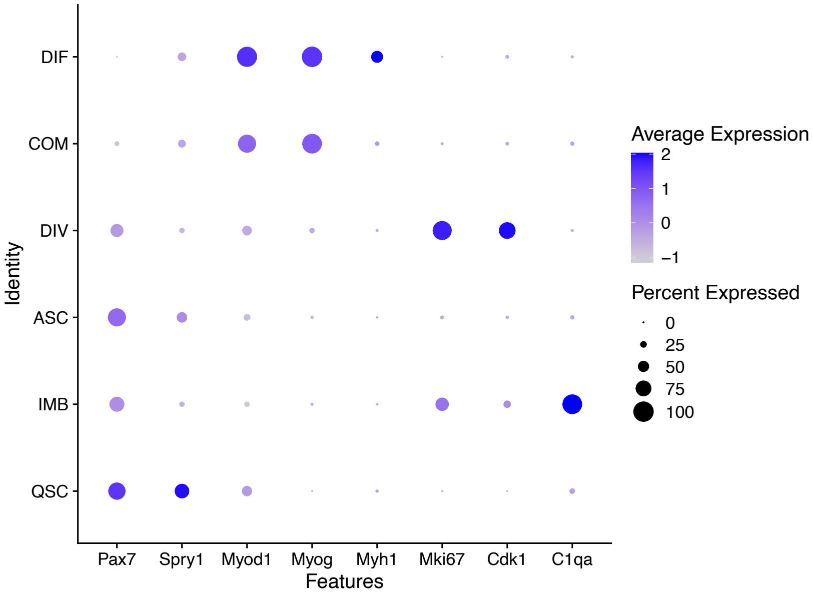

B

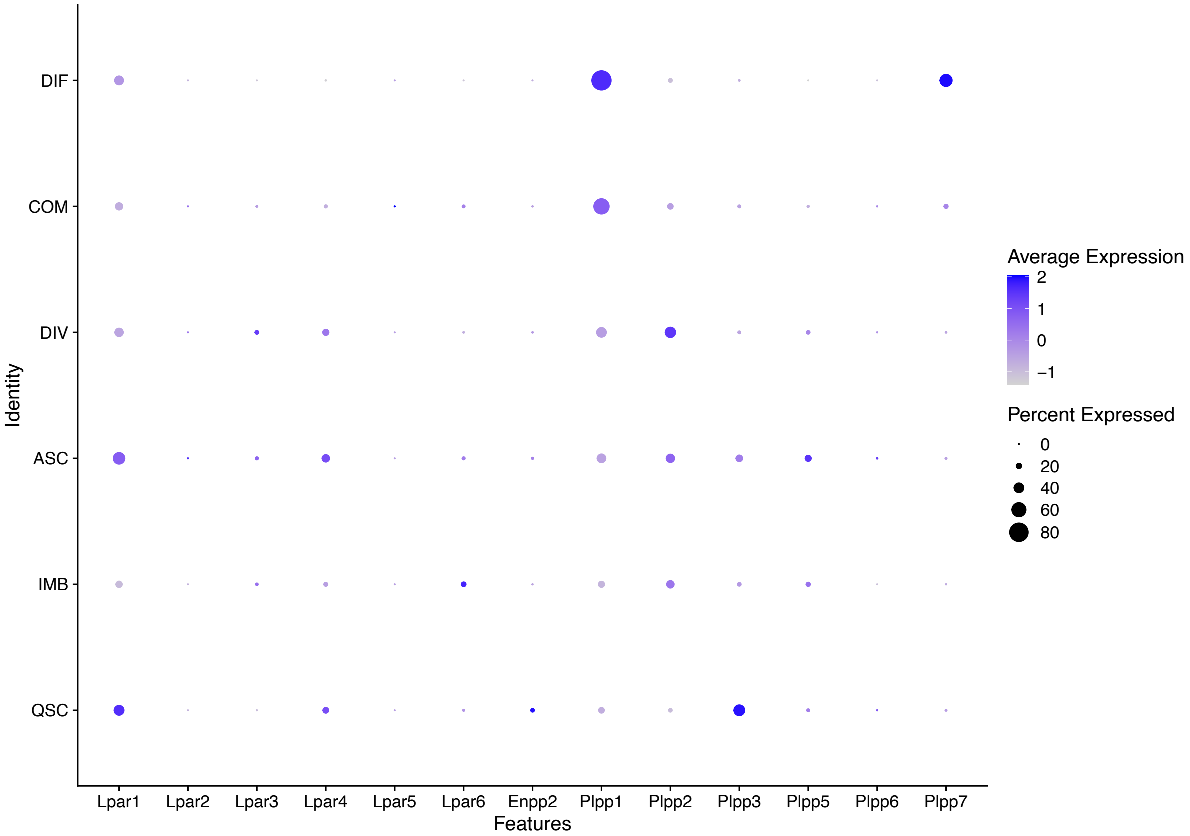

Supplementary figure 8. Gene expression dynamics of the ATX-LPAR-PLPP axis in muscle stem cells. (A) Dot plot showing gene expression levels of 8 differentially expressed genes between MuSC subpopulations. (B) Dot plot showing gene expression levels of LPAR, Enpp2, and Plpp family members in distinct MuSC subpopulations.
