# Supplementary material for: Single-cell transcriptome dynamics of the autotaxin-lysophosphatidic acid axis during muscle regeneration reveal proliferative effects in mesenchymal fibro-adipogenic progenitors
Source: Front Cell Dev Biol. 2023 Feb 23;11:1017660. doi: 10.3389/fcell.2023.1017660 (PMC9996314; doi:10.3389/fcell.2023.1017660)
Supplement: Supplementary file 3 [file DataSheet4.PDF]

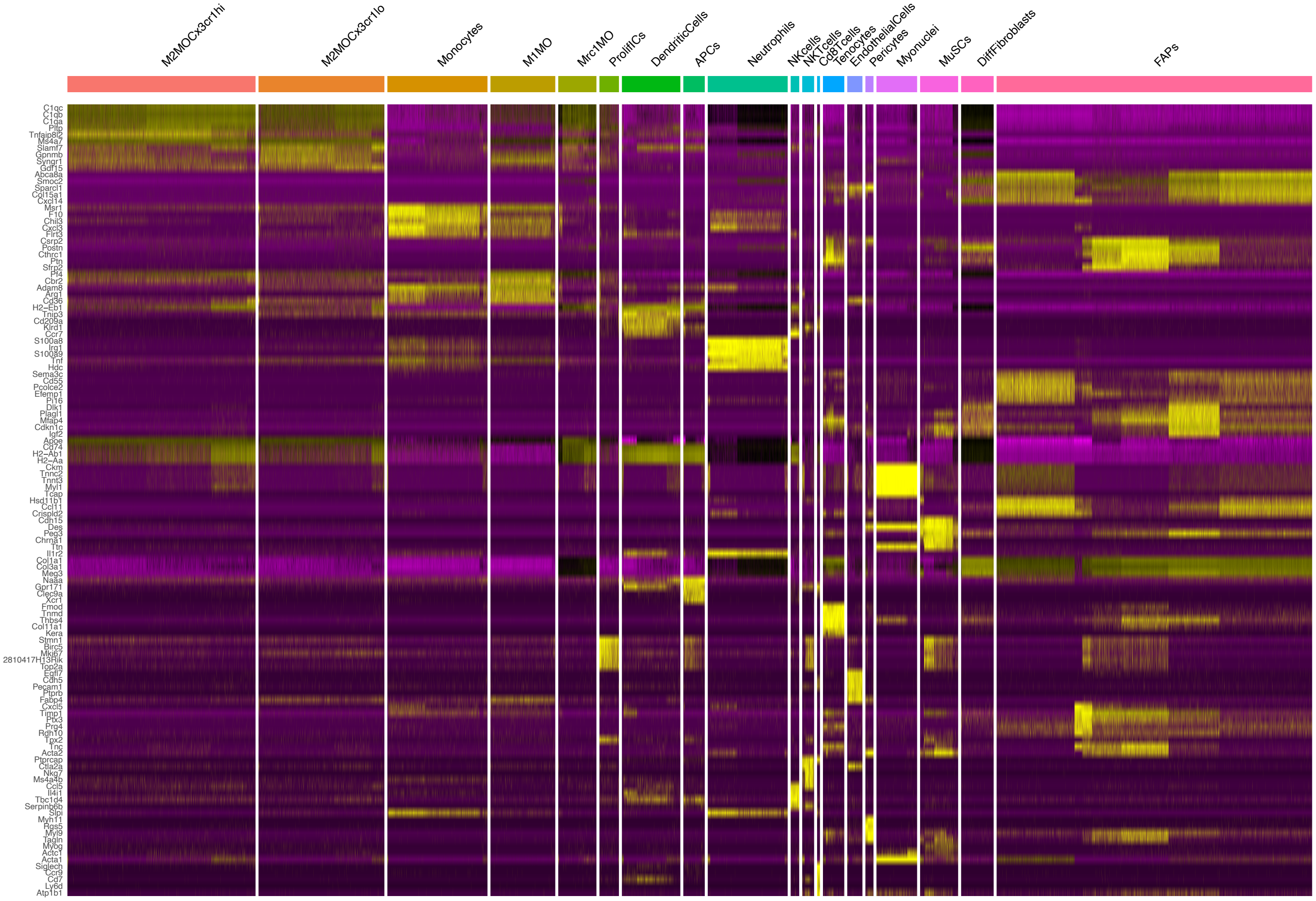

Supplementary figure 4. Heat map plot of top 10 upregulated genes and corresponding annotated cell populations. (A) Heat map plot showing distinct and major grouped cell populations across skeletal muscle homeostasis and regeneration. Cell populations and subpopulations were ordered based on the *top 10* expressed genes in each cluster subset shown in Supplementary figure 3. Please, note a high degree of transcriptomic variation among the FAPs subcluster.
